# Atlanta Medical College

**Published:** 1857-06

**Authors:** 


### ATLANTA MEDICAL COLLEGE.

This Institution opened on the 1st Monday in May, with an interesting Introductory, by Prof. J. Boring, which will be found in this number of our Journal; a good class is already in attendance—larger than last year at the same period we are assured by the Dean, whose calculation is, that there will be an increase in the class of 1857, of twenty-five per cent., over that of the last Session.

The facilities for teaching in various ways have been very considerably increased by late additions made to the appliances of the College, upon the return of Prof. Westmoreland from Europe, and the completion and reception of the remainder of the chemical Apparatus.

In addition to this, we have, for the first time, occupied our own building, which is now sufficiently completed for all practical purposes, and is acknowledged by all to be admirably adapted to the purpose for which it was designed—the dissecting room we would particularly mention as most complete, and amply filled, as we are assured, with fine anatomical material, and, altogether, we can say, we think without much boasting, that our arrangements for teaching medicine are not to be despised, if we *do* hold our sessions in the *summer*, to which some are so bitterly opposed.

Notwithstanding what we consider *good* advantages for teaching the Science of Medicine offered by the Atlanta Medical College, we are, as heretofore, *progressive*, and, at a proper time, will doubtless be ready to enter into any practical, feasible and *common sense* extension of facilities and requisitions for graduation, that may be generally adopted. As to the advice we have occasionally received of late (since we have grown up to be worthy of notice) from those who *pretend* to have a higher standard, all we have to say, is, gentlemen, we are very much obliged to you for your kind interest in our welfare, but consider ourselves entitled to the *right*, if not competent to the task of taking care of ourselves, and shall pursue the course dictated by our sense of right and propriety.

